# Drinking before and after pregnancy recognition among South African women: the moderating role of traumatic experiences

**DOI:** 10.1186/1471-2393-14-97

**Published:** 2014-03-05

**Authors:** Karmel W Choi, Laurie A Abler, Melissa H Watt, Lisa A Eaton, Seth C Kalichman, Donald Skinner, Desiree Pieterse, Kathleen J Sikkema

**Affiliations:** 1 Department of Psychology and Neuroscience, Duke University, Box 90086, Durham, NC 27708, USA; 2 Duke Global Health Institute, Duke University, Durham, NC, USA; 3 Department of Psychology, University of Connecticut, Storrs, CT, USA; 4 Stellenbosch University, Unit for Research on Health and Society, Tygerberg, South Africa

**Keywords:** South Africa, Pregnancy, Fetal alcohol spectrum disorder, Drinking, Trauma, Childhood abuse, Intimate partner violence

## Abstract

**Background:**

South Africa has one of the world’s highest rates of fetal alcohol spectrum disorder (FASD) and interpersonal trauma. These co-occurring public health problems raise the need to understand alcohol consumption among trauma-exposed pregnant women in this setting. Since a known predictor of drinking during pregnancy is drinking behavior before pregnancy, this study explored the relationship between women’s drinking levels before and after pregnancy recognition, and whether traumatic experiences – childhood abuse or recent intimate partner violence (IPV) – moderated this relationship.

**Methods:**

Women with incident pregnancies (N = 66) were identified from a longitudinal cohort of 560 female drinkers in a township of Cape Town, South Africa. Participants were included if they reported no pregnancy at one assessment and then reported pregnancy four months later at the next assessment. Alcohol use was measured by the Alcohol Use Disorders Identification Test (AUDIT), and traumatic experiences of childhood abuse and recent IPV were also assessed. Hierarchical linear regressions controlling for race and age examined childhood abuse and recent IPV as moderators of the effect of pre-pregnancy recognition drinking on post-pregnancy recognition AUDIT scores.

**Results:**

Following pregnancy recognition, 73% of women reported drinking at hazardous levels (AUDIT ≥ 8). Sixty-four percent reported early and/or recent exposure to trauma. While drinking levels before pregnancy significantly predicted drinking levels after pregnancy recognition, t(64) = 3.50, p < .01, this relationship was moderated by experiences of childhood abuse, B = -.577, t(60) = -2.58, p = .01, and recent IPV, B = -.477, t(60) = -2.16, p = .04. Pregnant women without traumatic experiences reported drinking at levels consistent with levels before pregnancy recognition. However, women with traumatic experiences tended to report elevated AUDIT scores following pregnancy recognition, even if low-risk drinkers previously.

**Conclusion:**

This study explored how female drinkers in South Africa may differentially modulate their drinking patterns upon pregnancy recognition, depending on trauma history. Our results suggest that women with traumatic experiences are more likely to exhibit risky alcohol consumption when they become pregnant, regardless of prior risk. These findings illuminate the relevance of trauma-informed efforts to reduce FASD in South Africa.

## Background

Heavy alcohol use during pregnancy is recognized for its deleterious impacts on fetal development and neonatal outcomes [1-3]. Children exposed to high concentrations of alcohol in utero tend to show a constellation of symptoms including low birth weight, developmental delays, neurocognitive deficits, and central nervous system abnormalities [4,5]. These symptoms constitute a range of conditions known as fetal alcohol spectrum disorders (FASD), with the most severe form being fetal alcohol syndrome (FAS). While the symptoms of FASD are often evident shortly after birth, individuals born with these conditions are also likely to experience lifelong consequences, including restricted educational achievement, poor social integration and reduced economic productivity [6,7].

One setting in which FASD is of great concern is South Africa, which has one of the world’s highest levels of alcohol consumption among drinkers [8]. Patterns of hazardous alcohol consumption have been observed among South African women [9], especially in the Western Cape, a wine-growing region where alcohol was historically paid as a form of wages for agricultural workers. In this region, community-based studies have also observed alarming rates of FASD [10,11], which are among the highest in the world [12]. Given the prevalence of FASD in this setting and its known consequences, there remains a need to understand factors that influence alcohol consumption during pregnancy, in order to identify further opportunities for intervention and prevention.

Research conducted in Western contexts suggests that a key predictor of drinking during pregnancy is a woman’s pre-pregnancy drinking profile [13,14]. A woman who drinks heavily is more likely to continue to drink during her pregnancy [15], perhaps due to habit and/or alcohol dependence. Notably, among South African women who report alcohol consumption, those who do drink tend to drink heavily [9], and so may be at increased risk to engage in alcohol consumption during pregnancy.

Another known predictor of hazardous drinking among women, though less explored among pregnant women, is trauma history. Experiences of childhood abuse, especially sexual abuse, have been linked to hazardous drinking among women in general [16,17]. In addition, experiencing violence in one’s adult relationships, or intimate partner violence (IPV), is also a risk factor for women’s alcohol use [18]. It is possible that exposure to interpersonal violence, whether childhood or recent, impacts women’s drinking behavior during pregnancy [19,20]. This is particularly relevant in South Africa given the high rates of interpersonal trauma and victimization that have been documented among South African women of childbearing age [21-23].

One question that remains unanswered in the literature is how women who drink heavily and/or regularly prior to pregnancy may alter their existing drinking patterns after recognizing pregnancy. Trauma history is one differential risk factor that may moderate the relationship between drinking before and after pregnancy recognition. Studies suggest that pregnancy is a psychologically stressful life transition for women [24,25] that may interact with previous stressors such as trauma to influence drinking trajectories. While there is limited literature on this phenomenon, our initial hypothesis is that a history of trauma exposure may make some women more likely to continue or even increase hazardous drinking behavior upon recognition of their pregnancy.

In this study, a longitudinal cohort of South African women was recruited from alcohol-serving venues to examine the relationship between drinking levels before and after pregnancy recognition, and whether a history of traumatic experiences (in recent intimate partner relationships and during childhood) would moderate this relationship. Examining risk factors that influence patterns of drinking behavior during the transition to pregnancy may provide novel insights for tailoring interventions among female drinkers to reduce hazardous alcohol consumption during pregnancy.

## Methods

### Setting

The study was conducted in Delft, a township located on the outskirts of Cape Town, South Africa. Delft was originally established in 1990 as part of a government project to integrate residents of Black (primarily Xhosa-speaking) and Coloured (primarily Afrikaans-speaking) ethnicities. According to a recent census [26], the unemployment rate in Delft and surrounding areas is high (49%), with a majority (66%) of households earning less than 3,200 rand (325 USD) each month. In addition, levels of education among Delft residents are low, with only 26% of adults having completed Grade 12 or beyond.

The study recruited participants from 12 alcohol-serving venues (six small informal venues called *shebeens* and six larger licensed *taverns*) selected from an initial list of 124 local venues identified by local informants. The 12 study venues (six with predominantly Black patrons and six with predominantly Coloured patrons) were chosen as representative establishments due to their diversity in terms of size, patronage, and location.

### Design

Data for this study came from a longitudinal cohort of 560 women assessed over a one-year period. This cohort has been described in a previous publication [27] and was not recruited solely for this study. Women were invited to participate in the cohort if they were observed to be regular patrons at one of the 12 study venues. Participants completed four assessments, each four months apart. Retention across the study was high, with 90.4% (506/560) of participants completing all four assessments. All assessments were conducted at a community-based study office using audio computer assisted interviewing (ACASI) in the participant’s preferred language (Xhosa, Afrikaans or English). Participants were compensated with grocery gift cards valued at 150 rand (approximately 20 USD) for each completed assessment. All study procedures were approved by the institutional review boards at Duke University, University of Connecticut, and Stellenbosch University.

For the present study, we extracted data from participants who became newly pregnant during the course of the cohort study. This sub-sample of incident pregnancies provided the opportunity to observe naturally occurring changes in alcohol consumption before and after pregnancy recognition. Our criterion for inclusion in this study was that women should have one assessment point when they reported not being pregnant, immediately followed by an assessment in which they reported pregnancy. We considered only women who had consecutive assessments, resulting in three possible pregnancy cohorts: women who were (1) not pregnant at the first assessment but pregnant at the second assessment; (2) not pregnant at the second assessment but pregnant at the third assessment; and (3) not pregnant at the third assessment but pregnant at the fourth assessment. We identified 66 women (12% of the overall sample) who fell into one of these three possible cohorts and hence were included in this study.

### Measures

#### Demographics and pregnancy

At each assessment, participants responded to basic questions about their age, race, marital status, educational level, and employment status. Participants were asked whether they planned to get pregnant in the coming year (yes/no) and to indicate whether they were currently pregnant at the time of assessment (yes/no).

#### Alcohol use

Participants completed the 10-item AUDIT (Alcohol Use Disorders Identification Test). The AUDIT measures a person’s frequency and quantity of drinking, and also provides a risk index of problematic drinking behavior. Each item on the AUDIT is rated from 0 to 4, and items are summed together to yield a continuous total score ranging from 0 to 40. An AUDIT score of 8 or higher is considered to be reflective of higher drinking risk and/or hazardous drinking patterns [28].

#### Recent intimate partner violence

Intimate partner violence (IPV) was assessed using a condensed version of the Revised Conflict Tactics Scales (CTS2) [29], with participants reporting any occurrence of various IPV events in the past four months. Emotional IPV was measured using two items on partner emotional abuse (“insulted or swore at you”, “threatened to hit or throw something at you”). Physical IPV was measured using four items on partner physical violence (“kicked, bit, or punched you”, “beat you”, “hit you with something”, “used a knife or gun on you”). Sexual IPV was measured using five items on coercive sexual experiences (“used force to make you have sex”, “used threats to make you have sex”, “made you have anal sex when you did not want to”, “made you use drugs or get drunk just so they could force you to have sex”, “forced to have sex with multiple men on one occasion”). We created dichotomous variables for each of the IPV subtypes, as well as an overall variable of “any recent IPV” (emotional, physical, and/or sexual).

#### Childhood abuse

Childhood abuse was assessed using items from the Childhood Trauma Questionnaire, Short Form (CTQ-SF) [30]. Childhood physical abuse, emotional abuse or neglect was measured using three items specific to treatment by family members (“hit by a family member so hard it left bruises or marks”, “called stupid, lazy or ugly”, “parents too drunk or high to take care of the family”). Childhood sexual abuse was measured using three items about unwanted sexual behavior (“tried to touch me in a sexual way or made me touch them”, “threatened me unless I did something sexual”, “forced me to have sexual intercourse”). We created dichotomous variables for each of the childhood abuse subtypes, as well as an overall variable of “any childhood abuse” (physical, emotional, neglect, or sexual).

### Analysis

We first extracted demographic and trauma history data as reported by participants at their pre-pregnancy assessment. Next, two separate AUDIT scores were created for each participant: (1) at the assessment prior to pregnancy recognition, henceforth referred to as the “pre-AUDIT” score; and (2) at the assessment after pregnancy recognition, henceforth referred to as the “post-AUDIT” score. Descriptive statistics were then performed to assess sample characteristics and drinking patterns.

The study’s outcome of interest was drinking behavior following pregnancy recognition, as measured by post-AUDIT scores. First, paired t-tests were conducted to examine any mean differences between pre-AUDIT and post-AUDIT scores. Next, bivariate regression analyses were run to examine the relationships between post-AUDIT scores and each predictor of interest: pre-AUDIT scores; any recent IPV; and any childhood abuse. Then, two hierarchical linear regressions (HLRs) were conducted to separately test the moderators of interest, recent IPV and childhood abuse. Both models controlled for the effects of race and age, which were entered in Step 1 of the HLR. These control variables were included as possible confounders because of their previous association with hazardous drinking patterns and also traumatic exposure [31-34]. The main effects of pre-AUDIT scores and traumatic exposure were then entered in Step 2, and the interaction term (pre-AUDIT x traumatic exposure) was entered in Step 3. The pre-AUDIT score variable was mean-centered prior to computing the interaction term, and this mean-centered term was also used to estimate the main effect. Age was also mean-centered for interpretation purposes. All HLR analyses were conducted using SPSS (SPSS IBM v. 21.0, Armonk, NY). Based on the results of each model, we created separate graphs (for recent trauma and childhood trauma) to visually depict the predicted relationship between pre-AUDIT and post-AUDIT scores, according to traumatic exposure. Finally, we calculated intraclass correlations to examine whether observed findings were potentially influenced by nesting of participants by venue, and carried out sensitivity analyses using generalized estimating equations to adjust for any effect of venue-level nesting and to calculate clustered robust standard errors for each parameter estimate.

As a follow-up analysis, similar HLR models were tested using the different IPV subtypes (physical, emotional, sexual) as moderators; patterns of interaction were visually consistent across the different models. Similarly, follow-up models were tested using the various childhood abuse subtypes (physical/emotional/neglect, sexual) as the moderators; the interactions terms were statistically non-significant, although the graphical patterns appeared to be similar. These findings are hence not presented in the following section.

## Results

### Description of the sample

#### Demographics

The sample characteristics are summarized in Table 1. The sample consisted of 66 women with a mean age of 29.0 (s.d. = 9.6). A slight majority (65%) identified themselves as Coloured, and 35% identified as Black. Participants reported low levels of education, with over 90% not having completed secondary school. Unemployment was also common among participants. Most women were unmarried and already had at least one child. The majority indicated at their pre-pregnancy time point that they were not planning to become pregnant within the next year, suggesting high rates of unintended pregnancy.

**Table 1:** Sample characteristics

**Variable**	**N (%)**
Age; mean (s.d.)	29.0 (9.6)
Race	
Black	23 (34.8%)
Colored	43 (65.2%)
Unmarried	45 (68.2%)
Has children	48 (72.7%)
Would attempt to get pregnant this year	50 (75.8%)
Unemployed	50 (75.8%)
Highest education reached	
Less than high school	22 (33.3%)
Some high school	39 (59.1%)
Completed high school (with diploma)	5 (7.6%)
Any traumatic experiences	42 (64%)
Any recent IPV	36 (54.5%)
Any childhood abuse	26 (39.4%)

Note: All variables were assessed prior to pregnancy recognition.

#### Trauma histories

Trauma exposures were common in this sample (Table 1). A majority of participants (64%, 42/66) reported exposure to some form of trauma, whether childhood abuse or recent IPV. Of these women, 14% reported experiencing only childhood abuse with no recent IPV, 38% reported only recent IPV with no history of childhood abuse, and 48% reported experiencing both early and recent experiences.

#### Drinking patterns

A summary of drinking patterns before and after pregnancy recognition is presented in Table 2. Prior to pregnancy recognition, 79% of the participants had AUDIT scores that met the criterion for hazardous drinking (AUDIT ≥ 8). The mean AUDIT score prior to pregnancy recognition was 14.8 (s.d = 7.87; range = 0–34), while the mean AUDIT score following pregnancy recognition was 12.7 (s.d = 7.72; range = 0–31). On average, there was a statistically significant reduction in drinking levels after pregnancy recognition, as compared to levels before pregnancy (*t* = -2.01, *p* = .048). The proportion of hazardous drinkers also decreased following pregnancy recognition, X^2^ (1, n = 66) = 12.3, p = .001. However, it is worth noting that the majority of women (73%) still drank at hazardous levels while aware of being pregnant. These drinking patterns are also presented by trauma exposure, comparing women with and without recent IPV (Table 3) and women with and without childhood abuse (Table 4). Overall, women with trauma exposure tended to have higher drinking scores than those without such a history.

**Table 2:** Drinking characteristics before and after pregnancy recognition

**Drinking variable**	**Pre-recognition n (%)**	**Post-recognition n (%)**	**Pre- and post- comparison**
AUDIT score; mean (s.d.)	14.8 (7.87)	12.7 (7.72)	t (65) = 2.02, p = .05*
Hazardous drinking (AUDIT ≥ 8)	52 (79.0%)	48 (72.9%)	X^2^(1, N = 66) = 12.3, p = .001**
*Frequency of drinking*			
Never	1 (1.5%)	6 (9.1%)	
Monthly or less	18 (27.3%)	18 (27.3%)	
2 – 4 times per month	24 (36.4%)	22 (33.3%)	
2 – 3 times per week	18 (27.3%)	16 (24.2%)	
Over 4 times per week	5 (7.6%)	4 (6.1%)	
*Drinks in a typical drinking day*	*n = 65*	*n = 60*	
1 – 2	19 (29.2%)	11 (18.3%)	
3 – 4	8 (12.3%)	19 (31.7%)	
5 – 6	19 (29.2%)	16 (26.7%)	
7 – 9	10 (15.4%)	5 (8.3%)	
10+	9 (13.8%)	9 (15%)	
*Binge/6 or more drinks per sitting*	*n = 65*	*n = 60*	
Never	11 (16.9%)	7 (11.7%)	
Less than monthly	14 (21.5%)	20 (33.3%)	
Monthly	11 (16.9%)	11 (18.3%)	
Weekly	28 (43.1%)	21 (35%)	
Daily or almost daily	1 (1.5%)	1 (1.7%)	

Note: ** for *p* < .01, * for *p* < .05.

**Table 3:** Pre-and post-pregnancy recognition drinking based on recent IPV exposure

**Drinking variable**	**No IPV (Total N = 30)**	**IPV (Total N = 36)**	**Statistical comparison**
Pre-AUDIT score	12.0 (SD = 7.4, range = 0-28)	17.3 (SD = 7.6, range = 2-34)	t (64) = -2.86, p = .006**
Post-AUDIT score	9.5 (SD = 6.5, range = 0-24)	15.4 (SD = 7.7, range = 0-31)	t (64) = -3.33, p = .001**
Pre-AUDIT ≥ 8 (hazardous drinking)	70% (N = 21)	86% (N = 31)	X^2^(1, N = 66) = 2.5, p = .011*
Post-AUDIT ≥ 8 (hazardous drinking)	57% (N = 17)	86% (N = 31)	X^2^(1, N = 66) = 7.2, p = .008**

Note: ** for *p* < .01, * for *p* < .05.

**Table 4:** Pre- and post-pregnancy recognition drinking based on childhood abuse exposure

**Drinking variable**	**No childhood abuse (Total N = 40)**	**Childhood abuse (Total N = 26)**	**Statistical comparison**
Pre-AUDIT score	12.7 (SD = 6.8, range = 0-25)	18.1 (SD = 8.4, range = 2-34)	t (64) = -2.87, p = 0.006**
Post-AUDIT score	10.5 (SD = 7.3, range = 0-29)	16.2 (SD = 7.2, range = 0-31)	t (64) = -3.13, p = 0.003**
Pre-AUDIT ≥ 8 (hazardous drinking)	78% (N = 31)	81% (N = 21)	X^2^(1, N = 66) = 0.1, p = .075
Post-AUDIT ≥ 8 (hazardous drinking)	60% (N = 24)	92% (N = 24)	X^2^(1, N = 66) = 8.29, p = .004**

Note: ** for p < .01.

Over 90% (n = 60) of the sample reported some alcohol consumption at their assessment following pregnancy recognition. Of those, a third (20/60) of the pregnant women reported drinking at least twice a week, and half (30/60) reported drinking five or more drinks on a typical day. A small group of women (15%, 9/60) reported having over 10 drinks in a typical day of drinking. In terms of binge-drinking, 37% (22/60) said they consumed 6 or more drinks in a single sitting on at least a weekly basis.

### Primary findings

#### Relationship between pre- and post-recognition drinking

Bivariate analyses revealed that drinking levels during pregnancy, as measured by post-AUDIT scores (reported after pregnancy recognition), were significantly predicted by pre-AUDIT scores (reported before pregnancy recognition), *B* = .39, *t* (64) = 3.50, *p* = .001. In addition, exposure to childhood abuse, *B* = 5.72, *t* (64) = 3.13, *p* = .003, and exposure to recent IPV, *B* = 5.92, *t* (64) = 3.33, *p* = .001 (both reported before pregnancy recognition), each significantly predicted post-AUDIT scores.

#### Recent IPV as a moderator

**Figure 1:**
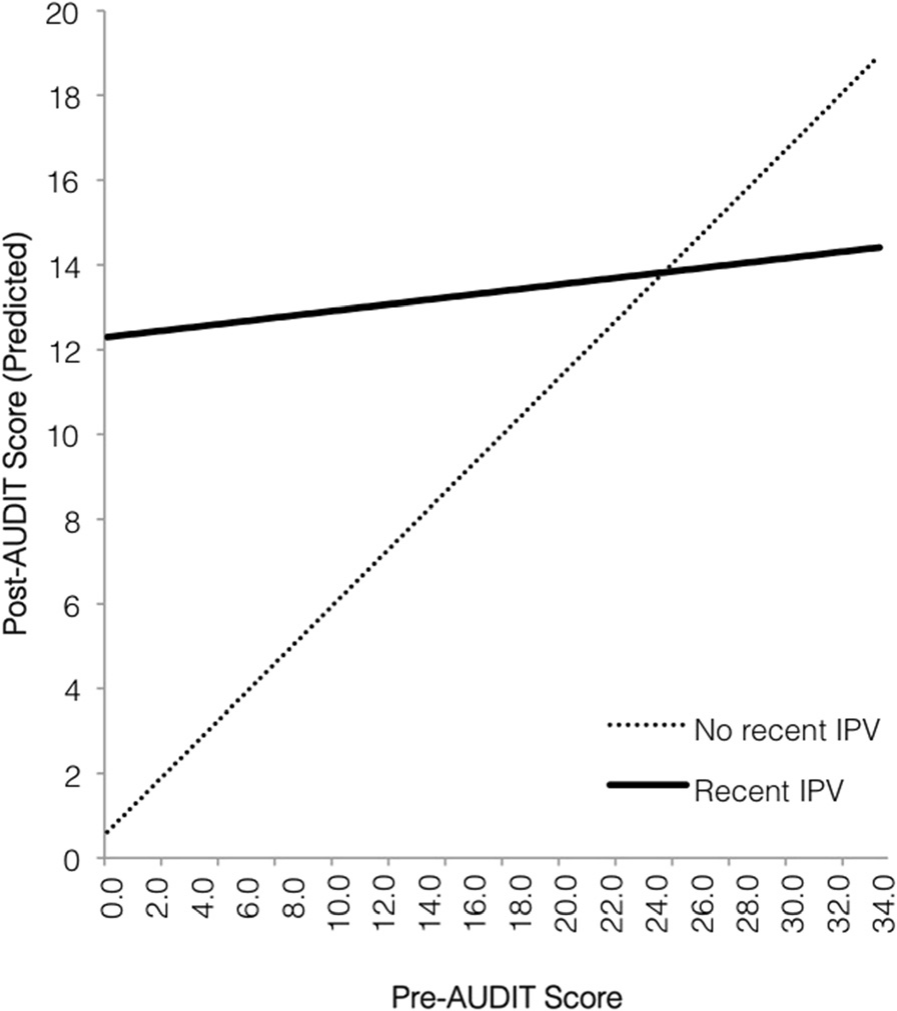
Predicted relationship between drinking levels before and after pregnancy recognition, for women with and without recent IPV.

In the model with recent IPV as a moderator (Table 5), the main effect of pre-AUDIT scores on the post-AUDIT scores was qualified by a significant interaction with recent IPV, *B* = -.477, *t* (60) = -2.16, *p* = .035. Race and age were controlled for in the model and were not significantly associated with the outcome. The interaction (Figure 1) indicated that for women with no recent IPV, AUDIT scores following pregnancy recognition were roughly proportional to AUDIT scores prior to pregnancy. However, for women with recent IPV, regardless of prior drinking risk, AUDIT scores following pregnancy recognition tended to fall above the cut-off for hazardous drinking.

**Table 5:** Main HLR analyses predicting post-AUDIT scores (after pregnancy recognition)

**Variables (by step entered)**	**Full model** ***B***	***p***
Moderator: Recent IPV		
1. Race	1.138	.056^✝^
Age (centered)	.028	.751
2. Pre-AUDIT score	.539	.002**
Any recent IPV	4.604	.010*
3. Pre-AUDIT x recent IPV	-.477	.035*
Moderator: Childhood abuse		
1. Race	.827	.163
Age	-.019	.831
2. Pre-AUDIT score	.572	.001**
Any childhood abuse	4.350	.017*
3. Pre-AUDIT x childhood abuse	-.577	.012*

Note: ** for *p* < .01, * for *p* < .05, ^✝^ for p < .10.

#### Childhood abuse as a moderator

**Figure 2:**
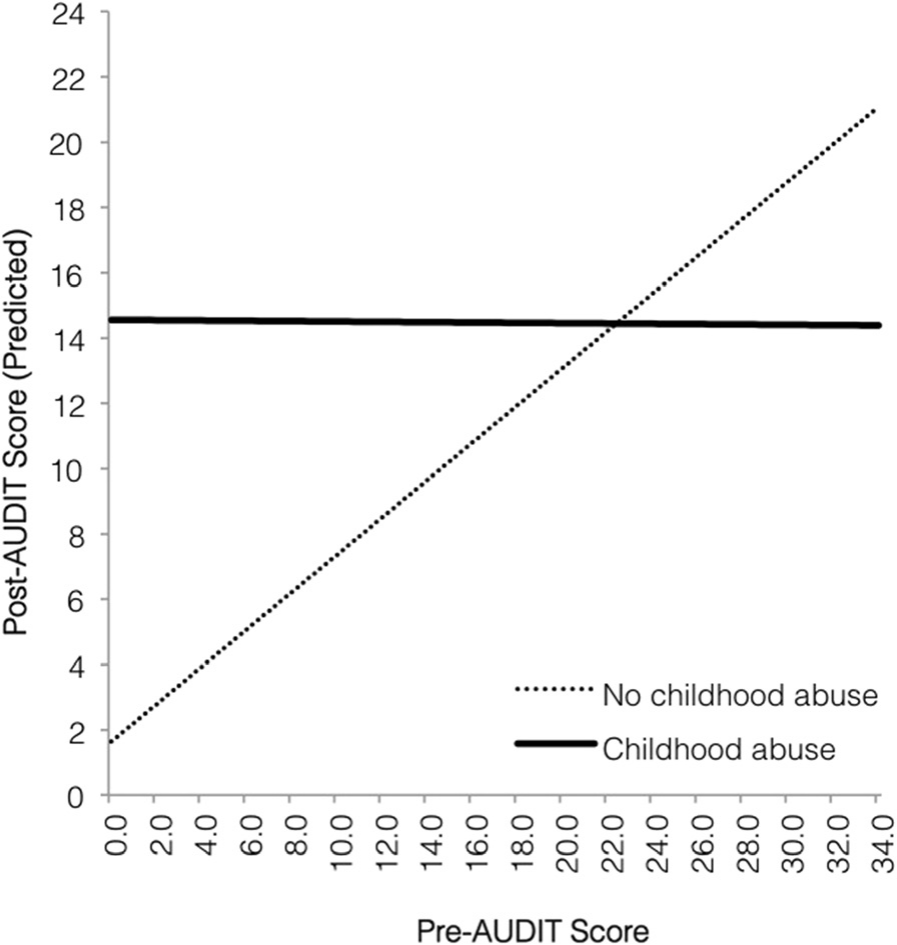
Predicted relationship between drinking levels before and after pregnancy recognition, for women with and without childhood abuse.

In the model with childhood abuse history as a moderator (Table 5), the main effect of pre-AUDIT scores on the post-AUDIT scores was qualified by a significant interaction with childhood abuse, *B* = -.577, *t* (60) = -2.58, *p* = .012. Again, race and age were controlled for in the model and were not significant predictors. The interaction (Figure 2) indicated a similar pattern in that women reporting no childhood abuse demonstrated proportional drinking behavior before and after pregnancy recognition, while women reporting childhood abuse tended to drink at hazardous levels during pregnancy, regardless of prior drinking behavior.

#### Exploring potential venue-level nesting

Given that participants were recruited from different venues, we explored whether there might be dependency in the data as a result of participants nested within the 12 venues. Dependency related to venue-level nesting was small, but notable (intraclass correlation = 0.08). However, this dependency was fully explained by race – such that when the individual-level race variable was included in a mixed model partitioning the drinking outcome into both individual-level and venue-level variances, the parameter estimate for the venue-level variance became zero. This suggests that dependency related to nesting at the venue level actually reflects the homogenous racial make-up of participants in the same venue, and that the venue-level differences are completely conditioned on race differences at the individual level, for which we had already adjusted in our earlier models. To corroborate this, our sensitivity analyses (data not shown) revealed the significant p-values from earlier models remained so, providing further support that venue-level nesting likely has minimal influence on the overall findings.

## Discussion

Drawing on a longitudinal cohort of female drinkers in South Africa, this study sought to prospectively examine the moderating effects of trauma history on drinking behavior before and after pregnancy recognition. As observed in prior studies in this setting [21,23], exposure to interpersonal violence, during childhood and/or in recent intimate relationships, was common among women. We also observed hazardous levels of alcohol use among these female drinkers, and high rates of unintended pregnancies. This is an at-risk population and highlights the importance of continued research at the intersection of trauma, pregnancy and alcohol use among South African women [35].

Similar to previous research [13,14], we found that women’s drinking levels before pregnancy were generally predictive of drinking levels during pregnancy. On average, participants significantly decreased their drinking after pregnancy recognition. This maps onto previous findings that women do tend to decrease their alcohol intake upon becoming pregnant [36]. However, it must be noted that a majority of participants reported drinking alcohol following pregnancy recognition, and most still drank at hazardous levels. This is also consistent with cross-sectional studies of drinking behavior among pregnant women in South Africa [37,38], and helps to contextualize the high rates of FAS and FASD documented in these communities [10,12].

Interestingly, our study suggests that female drinkers may be less likely to decrease their drinking during their transition to pregnancy if they have a history of trauma. Following pregnancy recognition, participants with prior experiences of IPV or childhood abuse tended to drink at elevated levels, regardless of prior drinking levels and even if they were low risk drinkers beforehand. On the other hand, participants with no trauma history seemed to drink proportionally to the levels that they did before pregnancy recognition, i.e., higher-risk drinkers continued to drink at hazardous levels, while lower-risk drinkers maintained their low drinking levels. Pregnancy recognition may trigger a shift in drinking behavior, differentially among women with and without a history of trauma. There is some evidence that women with a history of past abuse are more likely than women without such a history to experience heightened depressive or post-traumatic symptoms during pregnancy [39]. Recognition of pregnancy may act as an additional stressor that interacts with the woman’s trauma history to increase distress and related drinking behavior [40].

One of this study’s key strengths was its longitudinal sample, which offers an ideal opportunity to prospectively examine behavior changes during the transition to pregnancy [41]. Existing studies on women's alcohol use during pregnancy have tended to take a retrospective approach to assess drinking behavior, asking women to report on pregnancy behaviors in the post-partum period and beyond [15,42], which increases the likelihood of recall bias. Moreover, a prospective design allows women to report their drinking levels and traumatic experiences before they are even pregnant, without being influenced by a desire for consistency with their current behaviors and experiences during pregnancy.

Despite the strengths of this study, it also had several limitations that should be mentioned. First, drinking behavior and trauma histories were assessed via self-report. Due to social desirability bias, our participants may not have disclosed the full extent of their trauma histories or drinking habits, especially given that alcohol consumption among women is still not considered acceptable in the broader community [43]. However, the high levels of drinking and trauma reported in this sample are comparable to those previously observed among women in this setting [23,44,45], which suggests either that our results are reflective of reality or that previous research has similarly underestimated the presence of drinking and trauma among South African women. Second, drinking levels were measured using the overall AUDIT, which encompasses questions about both current and general drinking behavior. However, we did see variability in AUDIT scores before and after pregnancy recognition, suggesting that these scores were sensitive to changes from assessment to assessment. Third, it must be noted that our study focused on the time of women’s pregnancy *recognition* rather than actual conception. While intervening around the time of pregnancy recognition is valuable from a public health standpoint, there may be some women who are late to pregnancy recognition and hence represent an important population for future research and intervention [46]. In addition, our analyses focused on the transition from before to shortly after pregnancy recognition, and may not reflect drinking behaviors throughout the course of pregnancy. However, women in our study were likely in their first or second trimester of pregnancy, which is known to be a hazardous window for alcohol exposure [47,48]. Fourth, we focused on the moderating effects of any early or recent trauma histories, and less so on the specific subtypes of abuse or violence (physical, sexual, emotional). Our graphical analyses suggest that the nature of the impact seems to be the same regardless of trauma type. Given our small sample size, however, we were not able to fully explore these overlaps. We also considered the moderating effects of childhood abuse and IPV separately, though there is a subset of women who have experienced both. Future research should explore the types of traumatic experiences that have the most impact, and whether a lifetime history of both childhood and recent traumas has additive effects, or the impact of childhood abuse is mediated through greater risk for recent IPV. Finally, our sample size was modest, given that our study relied on naturalistic incident pregnancies in a community-based cohort of women. To our knowledge, it is the first effort to explore changes in drinking patterns among women already identified as heavy drinkers, an important target for prevention and intervention in any setting. Follow-up research using a larger, population-based sample could help to bolster and expand these preliminary findings.

The findings from our study suggest there are two groups of pregnant women that require special attention with regard to FASD prevention: (1) women who were already drinking at hazardous levels before pregnancy, and (2) women who report a history of trauma, regardless of how much they were drinking prior to pregnancy. In addition to general screening of substance use, trauma-informed care [49] in primary care settings is essential to both identify and intervene with South African women of childbearing age who have experienced trauma and hence may be at greater risk for adopting or maintaining hazardous drinking behavior during pregnancy. Once identified, these women might receive targeted interventions – both in the preconception period as well as throughout the antenatal period – such as motivational interviewing for alcohol use, psychological treatment for past trauma, and/or support for current stressors that might interact with past trauma to exacerbate drinking behavior during the transition to pregnancy.

## Conclusions

In conclusion, while prior studies have established that trauma history is a predictor of drinking behavior among pregnant women [19], [20], this study is the first to demonstrate that trauma history can also alter the relationship between drinking behavior before and after pregnancy recognition. These potential changes in drinking behavior are especially important to understand and target among female drinkers with a known history of alcohol consumption. Further insight into how traumatic experiences influence changes in drinking before and after pregnancy could help inform interventions to mitigate risky drinking trajectories during the transition to pregnancy, and reduce the burden of FASD in this setting.

## Competing interests

The authors declare that they have no competing interests.

## Authors’ contributions

All authors made substantive contributions to the work presented in this paper. KWC, LAA and MHW developed the concept for this paper, while KJS, DS and SCK conceived the longitudinal cohort design from which this present study was conducted. DP and DK coordinated the field-based acquisition of assessment data. KWC carried out the statistical analyses closely with LAA. Statistical results were interpreted with input from MHW, KJS, and LAE. KWC drafted the manuscript with substantial assistance from LAA and MHW. LAE, KJS, DS, DP, SCK reviewed and revised the manuscript critically for intellectual content. All co-authors provided final approval of the version to be published.

## Pre-publication history

The pre-publication history for this paper can be accessed here:

http://www.biomedcentral.com/1471-2393/14/97/prepub
